# Development of a Predictive Model for Optimization of Embryo Transfer Timing Using Blood-Based microRNA Expression Profile

**DOI:** 10.3390/ijms25010076

**Published:** 2023-12-20

**Authors:** Ming-Jer Chen, An Hsu, Pei-Yi Lin, Yu-Ling Chen, Ko-Wen Wu, Kuan-Chun Chen, Tiffany Wang, Yu-Chiao Yi, Hsiao-Fan Kung, Jui-Chun Chang, Wen-Jui Yang, Farn Lu, Hwa-Fen Guu, Ya-Fang Chen, Shih-Ting Chuan, Li-Yu Chen, Ching-Hung Chen, Pok Eric Yang, Jack Yu-Jen Huang

**Affiliations:** 1 Division of Reproductive Endocrinology and Infertility, Department of Obstetrics Gynecology & Women’s Health, Taichung Veterans General Hospital, Taichung 40764, Taiwan; mingjerchen@gmail.com (M.-J.C.); yjyihtw@yahoo.com.tw (Y.-C.Y.); eminem4389@gmail.com (H.-F.K.); r.juichun@gmail.com (J.-C.C.); hfku@vghtc.gov.tw (H.-F.G.); sylvan159x@gmail.com (Y.-F.C.); smile7966@hotmail.com (S.-T.C.); liyu0724@hotmail.com (L.-Y.C.); 2 Inti Labs, Hsinchu 30261, Taiwan; bernett@intilabs.com (A.H.); ivylin@intilabs.com (P.-Y.L.); yulingchen@intilabs.com (Y.-L.C.); kowenwu@intilabs.com (K.-W.W.); kentchen@intilabs.com (K.-C.C.); tiffany@intilabs.com (T.W.); 3 Taiwan IVF Group Center for Reproductive Medicine and Infertility, Hsinchu 30274, Taiwan; molinda11@gmail.com (W.-J.Y.); farn.lu@gmail.com (F.L.); icryoegg@gmail.com (C.-H.C.); 4 Department of Obstetrics & Gynecology, Stanford University, Stanford, CA 94305, USA

**Keywords:** endometrial receptivity, non-invasive biomarkers, blood, cell-free microRNA, next-generation sequencing

## Abstract

MicroRNAs (miRNAs) can regulate the expression of genes involved in the establishment of the window of implantation (WOI) in the endometrium. Recent studies indicated that cell-free miRNAs in uterine fluid and blood samples could act as alternative and non-invasive sample types for endometrial receptivity analysis. In this study, we attempt to systematically evaluate whether the expression levels of cell-free microRNAs in blood samples could be used as non-invasive biomarkers for assessing endometrial receptivity status. We profiled the miRNA expression levels of 111 blood samples using next-generation sequencing to establish a predictive model for the assessment of endometrial receptivity status. This model was validated with an independent dataset (*n* = 73). The overall accuracy is 95.9%. Specifically, we achieved accuracies of 95.9%, 95.9%, and 100.0% for the pre-receptive group, the receptive group, and the post-respective group, respectively. Additionally, we identified a set of differentially expressed miRNAs between different endometrial receptivity statuses using the following criteria: *p*-value < 0.05 and fold change greater than 1.5 or less than −1.5. In conclusion, the expression levels of cell-free miRNAs in blood samples can be utilized in a non-invasive manner to distinguish different endometrial receptivity statuses.

## 1. Introduction

In recent years, identifying possible displacement or disruption of the window of implantation (WOI) in patients with recurrent implantation failure (RIF) is suggested as a critical process to increase the overall implantation rate during in vitro fertilization (IVF) treatment cycles. Several associated assays and platforms have been developed for assessing the window of implantation [1,2,3,4]. Using endometrial tissue samples has been the most common approach up to now, but it is an invasive procedure for endometrial receptivity analysis. The possibility of using bodily fluids (for example, uterine fluids and blood samples) has been explored as a potentially promising non-invasive sample type for endometrial receptivity analysis [5,6,7,8,9]. Local injury to the endometrium can have a negative impact on implantation [10]. Additionally, it is impossible to perform endometrial tissue analysis to guide implantation timing in the same cycle. Therefore, developing a non-invasive diagnostic tool to accurately predict the WOI is an important step in the field of reproductive medicine.

MiRNAs are non-coding ribonucleic acids (RNAs) 15–40 nucleotides long found in humans and other multicellular organisms and are involved in gene regulation. More than 2500 miRNAs have been discovered in the human genome, some of which have been widely reported as gene regulators on post-transcriptional levels involved in various biological processes such as gametogenesis; embryogenesis and the quality of sperms, oocytes, and embryos [11,12]; and diseases such as endometriosis, endometritis, and endometrial cancer [13,14,15]. MiRNAs can also regulate the expression of genes involved in the establishment of WOI [11,16]. MiR-30b was involved in cyclic remodeling of the endometrium, including endometrial maturation to the receptive state [17]. Decreased expressions of miR-181 and miR-223-3p are detrimental to initiating implantation, since they lower the expression of LIF, a promising marker of implantation, and impeded implantation [18,19]. Additionally, dysregulation of miRNAs could contribute to the RIF experienced by infertile patients. Elevated levels of miR-21 and miR-22 reduced the expression levels of KLF12 and TIAM/RAC1, respectively, which are important in early stromal cell decidualization [20,21].

MiRNA can be secreted into extracellular fluid and circulation, providing a more stable type of biomarker [22,23,24]. In comparison to messenger RNAs (mRNAs), circulating miRNAs are more resistant to endogenous ribonuclease activity [25]. Plasma/serum miRNAs have been postulated as useful biomarkers for a variety of conditions, such as cancer, cardiovascular disorders, immune diseases, inflammatory diseases, Alzheimer’s disease and infectious diseases [26,27,28,29]. In this study, we use microRNAs (miRNAs) as biomarkers in blood samples to systematically construct a prediction model for assessing the window of implantation. The resulting non-invasive diagnostic assay is the first of its kind for predicting the status of endometrial receptivity during hormone replacement therapy (HRT) for the purpose of frozen–thawed embryo transfer.

## 2. Results

### 2.1. Profiling of the miRNA Expression Level in Plasma Sample

A total of 111 blood samples that had been confirmed for endometrial status through MIRA endometrial receptivity testing and successful implantation result were used for model development. The timepoint of blood collection coincided with the timepoint of endometrial tissue sample collection. The dataset consisted of 30 samples in the pre-receptive phase (Pre), 75 samples in the receptive phase (Rec), and 6 samples in the post-receptive phase (Post) (Supplemental Table S1, the prediction model building dataset).

To systematically identify the expression profile of miRNAs in the blood samples, we employed NGS (next-generation sequencing) as the detection tool in this study. The complete experimental and data analysis workflow is illustrated in Supplemental Figure S1. After the completion of miRNA extraction and small RNA library construction, NGS was performed. Upon sequence alignment, miRBase was utilized for annotation to identify all possible miRNAs. The sequencing results of the 111 samples are presented in Supplemental Table S2. The average sequencing depth was 8,000,795x, and the average read counts of detectable miRNAs was 395,433 reads, accounting for an average proportion of 4.8% of the total sequencing reads. The average number of detectable miRNAs was 135 (Supplemental Table S2).

### 2.2. Establishment of a Prediction Model to Determine the Endometrial Receptivity Status

**Figure 1:**
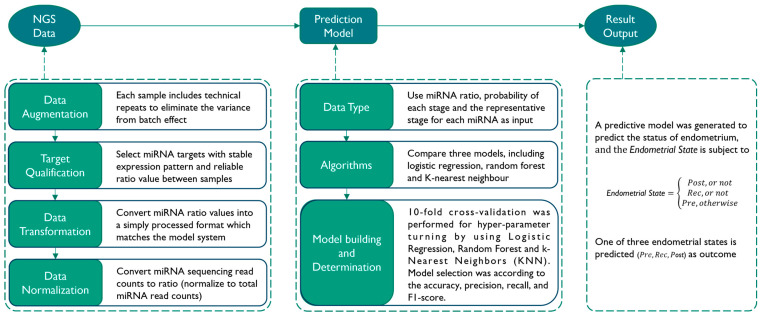
Workflow of prediction model building. The process of prediction model building consists of three main steps. The first step is to preprocess the NGS data before model building, which involves data augmentation, transformation, and normalization. The second step is the model building process, where different data types and algorithms are tested simultaneously, and the best model is selected based on its performance. The third step involves verifying the results of the testing data output.

Based on the miRNA expression profiles of 111 blood samples, we next attempted to establish a prediction model that was able to distinguish three stages of endometrial status (Pre, Rec, and Post). The data analysis process and the factors taken into consideration are described in Figure 1. Briefly, the analysis combined a series of bioinformatics analysis, data processing, model training, and evaluation steps to build an in-house machine learning pipeline for classifying specimens into different endometrial receptivity statuses. We performed 10-fold cross-validation for hyper-parameter turning by using Logistic Regression, the Random Forest Classifier, and k-Nearest Neighbors (KNN). A prediction model was selected by analyzing the results from different combinations of the factors and algorithms. The overall accuracy of this prediction model is 91.9% (Table 1).

### 2.3. Validation of the Prediction Model

To validate the performance of the prediction model, we recruited another set of blood samples, including 3 samples in Pre, 66 samples in Rec, and 4 samples in Post (Supplemental Table S1, validation dataset) determined by MIRA and which have successful implantation results. After analyzing all 73 samples using the prediction model, the overall accuracy is 95.9%. Specifically, we achieved accuracies of 95.9%, 95.9%, and 100.0% for the pre-receptive group, the receptive group, and the post-respective group, respectively (Table 2).

### 2.4. Identification of Differentially Expressed miRNAs among Different Endometrial Receptivity Statuses

**Figure 2:**
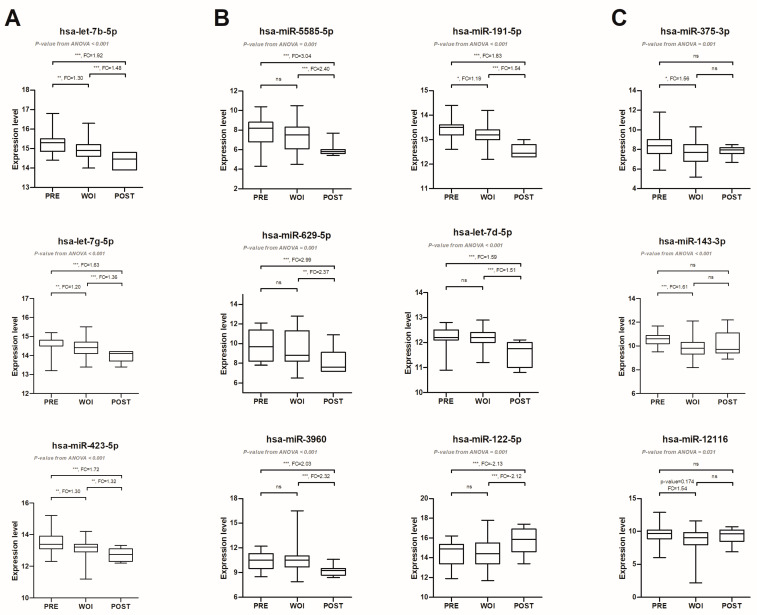
Differentially expressed miRNAs among different endometrial receptivity statues. According to the expression levels of miRNAs in different statuses, miRNAs can be classified into three conditions: miRNAs expressed with decreasing expression levels from Pre group to Rec group and further to Post group (**A**); miRNAs in the Post group exhibited differential expression levels compared to the other two groups (**B**); and differentially expressed miRNAs between Pre and Rec groups (**C**). The expression level of each miRNA is determined by first obtaining the ratio of miRNA reads to total miRNA reads, and then multiplying the ratio by 1,000,000. Finally, the resulting value is calculated by taking a log2 transformation. For each box plot, the medians are shown as a black horizontal line in the box, where the box represents the upper and lower quartiles. The upper and lower whiskers show the maximum and minimum, respectively. Significant post hoc comparisons are represented by asterisks. * *p*-value < 0.05; ** *p*-value < 0.01; *** *p*-value < 0.001; ns: no significance, FC: fold change.

We conducted an analysis to identify differentially expressed miRNAs in the blood among different endometrial receptivity statuses (Supplemental Table S3). The expression patterns varied across different endometrial receptivity statuses. First, we observed a set of miRNAs, including hsa-let-7b-5p, hsa-let-7g-5p, and hsa-miR-423-5p, with decreasing expression levels from pre-receptive to receptive to post-receptive endometrial status (Figure 2A).

Secondly, there are specific stages where distinct miRNAs exhibit either higher or lower expression levels. For instance, in the Post group, miRNAs such as hsa-miR-5585-5p, hsa-miR-629-5p, hsa-miR-3960, hsa-miR-191-5p, and hsa-let-7d-5p had significantly lower expression levels compared to the Pre and Rec groups. On the other hand, hsa-miR-122-5p had significantly higher expression levels in the Post group compared to the Pre and Rec groups (Figure 2B). Moreover, miRNAs in the Pre group exhibited significantly higher expression levels than the Rec group, including hsa-miR-375-3p, hsa-miR-143-3p, and hsa-miR-12116 (Figure 2C). Overall, changes in miRNA expression levels indicate a complex regulation of gene expression during the preparation of the endometrium for implantation.

## 3. Discussion

Detecting endometrial receptivity has been shown to improve the success rate of embryo implantation [30]. Developing non-invasive methods to replace the existing invasive methods for detecting endometrial receptivity would make the process safer and more convenient. In this study, we utilized the expression profiles of miRNAs from 111 blood samples which have successful implantation results to establish a predictive model for endometrial receptivity status. We also validated the model’s performance using a validation dataset consisting of 73 blood samples. The model achieved a 95.9% accuracy in predicting endometrial receptivity status.

A limitation of this study is that the validation dataset had a smaller number of Pre samples (n = 3) and Post samples (n = 4). Therefore, the results may demonstrate less significant analytical performance. For example, the sensitivity and positive predictive value of the Pre group are 66.7% and 50.0%, respectively (Table 2). Even though the analytical performance for the Post group is 100.0%, further validation with a larger number of samples is necessary to properly assess the performance of this model. Despite the limited number of samples, the experimental and model building process shows the feasibility of using miRNA expression patterns in blood samples as a diagnostic marker for assessing receptivity status in the endometrium. Additionally, in order to encompass the different IVF treatment protocols used [31], it would also be beneficial to collect plasma samples from subjects undergoing natural cycles to further optimize the prediction model. With the addition of clinical samples collected from natural cycles, we can further confirm any differences in miRNA expression between natural and HRT cycles, allowing more flexibility in use of the algorithm.

Additionally, we identified several miRNAs that exhibited significant expression differences among the pre-receptive, receptive, and post-receptive groups (Figure 2) in the blood. Among them, three miRNAs belonged to the let-7 family. One of these miRNAs, hsa-let-7g-5p, has a known target gene called IGF2R (insulin-like growth factor 2 receptor). IGF2R plays a role in pregnancy establishment and maintenance, and hsa-let-7g-5p disrupts decidualization by regulating the expression of IGF2R [32]. In HRT cycles preparing for frozen embryo transfer, progesterone is commonly administered to control the timing of endometrial maturation for facilitating embryo implantation. Progesterone could also regulate the expression of certain miRNAs in the endometrial epithelium tissue, including has-miR-143-3p. has-miR-143-3p could inhibit the proliferation of endometrial epithelial cells by regulating the expression of cyclin D2, thus affecting the growth of the endometrial epithelium [33]. Involvement of hsa-miR-191-5p starts in the initial stage of implantation and a bidirectional molecular communication between the blastocyst and endometrium, which aids in establishing the initial stage of implantation [34]. hsa-miR-191-5p and other miRNAs could impact the establishment of the initial stage of implantation through disrupting cell cycle regulation and proliferation of endometrial epithelial cells [34].

When developing a predictive model, it is crucial that markers can be reliably detected and exhibit significant differences in expression levels between the control group and target group. The presence of known biological functions of a marker is relatively less important. Therefore, besides miRNAs, there are other types of small non-coding RNAs that can be stably detected in blood samples. These alternative RNA types may also serve as effective markers. One such example is PIWI-interacting RNA (piRNA), which may have a novel and effective diagnostic role in lung adenocarcinoma, digestive system cancer, and breast cancer [35,36,37,38]. Our study used NGS to detect miRNA expression. Supplemental Table S2 shows that, on average, miRNAs accounted for approximately 5% of the total sequencing reads. Therefore, a significant proportion of small RNAs remain unexplored, providing a promising avenue for further investigation to determine their potential as effective markers for prediction model building.

In addition to the endometrial receptivity status, the success rate of embryo implantation can also be influenced by other uterine disorders. Conditions such as endometriosis, chronic endometritis, and endometrial cancer have been linked to compromised outcomes [13,14,39]. MiRNAs in blood sample have the potential to detect these diseases [13,39,40]. In the future, it may be possible to incorporate the detection of disease-related miRNAs into the predictive model mentioned in this study. By integrating the assessment of these markers, a single test could potentially identify multiple factors that affect the outcome of embryo transfer. As a result, proper treatment can be prescribed for the patients based on the detected factors [41]. Moreover, if the quantity of miRNAs can be directly and stably monitored in vivo [42], the test would greatly increase the success rate of individuals undergoing IVF by measuring effectiveness of the treatment.

As the testing time required for an endometrial receptivity assay is usually more than a week, personalized embryo transfer is typically performed in a subsequent cycle, separated from the sample collection cycle. If the testing time could be shortened to one or two days, there would be an increasing possibility of completing the entire treatment within the same cycle. This could potentially allow for the option of personalized fresh embryo transfer, decreasing the cost associated with embryo cryopreservation.

In recent years, there has been a rapid development of artificial intelligence (AI) with significant applications in various fields, including assisted reproduction technologies (ART). AI has played an important role in ultrasound monitoring, embryo selection, and male infertility assessment [43,44] as automation and accuracy continue to improve. In this study, we utilized machine learning/artificial intelligence to identify biological and clinical signatures that distinguish the optimal timing for embryo implantation. In the future, artificial intelligence may become the most helpful diagnostic tool in IVF labs or reproductive centers. It is important to consider additional legal and ethical policies as molecular tests advance in reproductive medicine to ensure patient protection. As personalized medicine becomes more prevalent in this field, further discussions are needed regarding the autonomy, confidentiality, privacy, and equity of patients after utilizing molecular tests during their IVF treatment cycle [45,46]. 

## 4. Materials and Methods

### 4.1. Study Population

The population under this retrospective study consisted of two cohorts (prediction model building dataset and validation dataset) of 184 total subjects with successful implantation results from May 2021 to October 2022. The inclusion criteria for the subjects were as follows: age between 21 and 45 years; absence of ovulatory disorders, endometriosis, myomas, polyps, or hydrosalpinx; body mass index (BMI) > 18.5 kg/m^2^; have at least one (including one) good frozen blastocyst for transfer. A good blastocyst was defined as grade 3BB and above (Gardner blastocyst grading system) [47]. Both endometrial tissue and peripheral blood samples were collected during their first hormone treatment cycle preparing for the frozen embryo transfer, 120 h after the beginning of intramuscular progesterone injection. To identify the stage of endometrial receptivity, the endometrial tissue was tested via a ready-to-use endometrial receptivity assay already on the market, MIRA^TM^ (Inti Labs, Hsinchu, Taiwan). In summary, the MIRA^TM^ test involves collecting endometrial tissue samples from the uterine cavity using a Pipelle catheter (Gynetics, Lommel, Belgium, Cat. No. #4164) after 120 ± 3 h of progesterone administration in an HRT cycle. Total RNAs are then isolated using miRNeasy Micro Kit (QIAGEN, Stockach, Germany, Cat. No. 217084). Subsequently, miRNA expression profiles are obtained and analyzed using the multiplex qPCR system PanelChip^®^ [2]. Finally, the endometrial receptivity status is determined based on the miRNA expression pattern using a predictive model established with the elastic-net regularized generalized linear model [48]. Embryo transfer was performed in the second hormone treatment cycle based on the result of the MIRA test. Blood samples from 184 subjects with successful implantation results were analyzed for miRNA expression pattern and prediction model establishment. This study was approved by the Institutional Review Board (IRB) of Taichung Veterans General Hospital (TCVGH) (IRB Number: SF21040A) and the Taiwan IVF Group (JIRB Number: 18-003-A-2), and study subjects were included only after written informed consent forms were obtained.

### 4.2. Plasma Sample Collection and Preparation

Peripheral blood samples (5–10 mL per subject) were obtained from subjects undergoing hormone treatment cycle. The blood sample was collected into EDTA tubes (BD, MIS, Canada, Cat. No. 367525) or Plasma Preparation Tubes (BD, MIS, Canada, Cat. No. 362788). After the blood samples were collected, the tubes were inverted at least five times and processed within 60 min. Each specimen was centrifuged at 1200 *g* for 10 min at room temperature to separate plasma from cells. The supernatant was transferred to new tubes and centrifuged at 12,000× *g* for 10 min. The plasma samples were subsequently transferred to new tubes and stored at −80 °C.

### 4.3. miRNA Extraction and Preparation

Plasma RNA was isolated from 200–600 μL of plasma with miRNeasy Serum/Plasma Advanced Kit (QIAGEN, Germany, Cat. No. 217204) following the manufacturer’s protocol. Subsequently, the plasma RNA was eluted in nuclease-free water. The concentration was quantified by using Qubit microRNA Assay Kit (Thermo Fisher Scientific, MA, USA, Cat. No. Q32880). For each sample, at least 10ng microRNA was used as input for the following library construction.

### 4.4. miRNA Library Construction and Sequencing

The miRNA sequencing library was constructed using the QIAseq miRNA Library Kit (QIAGEN, Germany, Cat. No. 331502). In brief, the miRNA sequencing library was prepared by the following steps: (1). 3′-adaptor ligation with pre-adenylated adaptor; (2). 5′-adaptor ligation with sequenced primers; (3). cDNA synthesis using reverse transcription primers with a unique molecular index (UMI) assigned to every miRNA molecule, allowing the identification of individual molecules; (4). cDNA cleanup; (5). PCR amplification using primers with sample barcode; (6). library cleanup. The quality of the library was checked using the 5200 Fragment Analyzer System (Agilent Technologies, CA, USA). The size of the library product is between 190 and 220 bps. The library was quantified by Qubit (Thermo Fisher Scientific, USA, Cat. No. Q32851), and the concentration must be more than 1 ng/μL for the following sequencing assay. The library was sequenced on the Illumina NextSeq 550 (Illumina, CA, USA) as per the manufacturer’s instructions.

### 4.5. NGS Data Analysis Pipeline

A NGS data analysis pipeline was built to analyze the data, with the following protocols: (1). Data were preprocessed by performing quality control on raw fastq data, including trimming adapter sequences, and removing low-quality reads using FastQC [49] and Trimmomatic [50]. After trimming low-quality (Q value < 20) ends from reads in addition to adapter removal, reads shorter than 17bps or longer than 55bps were discarded. (2). Alignment of processed reads to Human Genome Assembly GRCh38 (hg38) reference genome and a specific set of small RNA sequences from miRBase was performed by using aligners such as Bowtie [51,52]. (3). Quantification of extracted reads from the data by mapping the aligned reads using samtools and obtained reference annotations using miRBase [53]. The read counts of each miRNA were used as the expression value for further data analysis.

### 4.6. Establishment of a Prediction Model

Data augmentation was first performed in silico via repeated random sampling of five million reads from the NGS data in duplication of the same specimen. Subsequently, a cut-off value of read counts greater than 20 is used to filter miRNA expression levels. To normalize the data, the total read counts of miRNAs is calculated by summing the read counts of filtered miRNAs via a data transformation step for each sample. The ratio of each miRNA in a sample is calculated by dividing each value in the selected miRNA read counts by miRNA total read counts. A 10-fold cross-validation was performed for hyper-parameter turning by using Logistic Regression, the Random Forest Classifier, and k-Nearest Neighbors (KNN) [54,55,56]. Detailed information for model building is available in the Supplemental Information.

### 4.7. Identification of Differentially Expressed miRNAs

Differences in miRNA abundance among different endometrial status groups were analyzed with ANOVA and post hoc Tukey HSD test. After transformation and normalization of the raw miRNA reads of each sample, a *p*-value < 0.05 and fold change >1.5 or <−1.5 was considered statistically significant.

## 5. Conclusions

In conclusion, our finding indicated that the expression profile of miRNAs from blood samples can be utilized as non-invasive biomarkers for distinguishing the different endometrial receptivity statuses, including pre-receptive, receptive, and post-receptive.

## 6. Patents

A patent has been filed as a provisional application.

## Figures and Tables

**Table 1:** Performance of prediction model building dataset.

		Known Endometrial Status			Sensitivity ^1^	Specificity ^2^	PPV ^3^	NPV ^4^	Accuracy ^5^
		Pre	Rec	Post					
Predicted result	Pre	21	0	0	70.0%	100.0%	100.0%	90.0%	91.9%
	Rec	9	75	0	100.0%	75.0%	89.3%	100.0%	91.9%
	Post	0	0	6	100.0%	100.0%	100.0%	100.0%	100.0%
						Overall Accuracy			91.9%

^1^ Sensitivity = True Positives/(True positives + False negatives). ^2^ Specificity = True negatives/(True negatives + False positives). ^3^ PPV, Positive predictive value or precision = True positives/(True positives + False positives). ^4^ NPV, Negative predictive value = True negatives/(True negatives + False negatives). ^5^ Accuracy = (True positives + True negatives)/(True positives + False positives + False negatives + True negatives).

**Table 2:** Performance of clinical validation dataset.

		Known Endometrial Status			Sensitivity ^1^	Specificity ^2^	PPV ^3^	NPV ^4^	Accuracy ^5^
		Pre	Rec	Post					
Predicted result	Pre	2	2	0	66.7%	97.1%	50.0%	98.6%	95.9%
	Rec	1	64	0	97.0%	85.7%	98.5%	75.0%	95.9%
	Post	0	0	4	100.0%	100.0%	100.0%	100.0%	100.0%
						Overall Accuracy			95.9%

^1^ Sensitivity = True Positives/(True positives + False negatives). ^2^ Specificity = True negatives/(True negatives + False positives). ^3^ PPV, Positive predictive value or precision = True positives/(True positives + False positives). ^4^ NPV, Negative predictive value = True negatives/(True negatives + False negatives). ^5^ Accuracy = (True positives + True negatives)/(True positives + False positives + False negatives + True negatives).

## Data Availability

The data presented in this study are available on request from the corresponding author. The data are not publicly available due to containing information that could compromise the privacy of research participants.

## Supplementary Materials

The following supporting information can be downloaded at: https://www.mdpi.com/article/10.3390/ijms25010076/s1.
