# Supplementary material for: Development of a Predictive Model for Optimization of Embryo Transfer Timing Using Blood-Based microRNA Expression Profile
Source: Int J Mol Sci. 2023 Dec 20;25(1):76. doi: 10.3390/ijms25010076 (PMC10779357; doi:10.3390/ijms25010076)
Supplement: Supplementary file 1 [file ijms-25-00076-s001.zip › ijms-2716971-supplementary.pdf]

Supplementary material

Supplemental Figure S1. Overview of the study design, including the number of samples used in the experimental design for model building and validation, experimental steps, and primary data analysis process.

Supplemental Table S1. Clinical characteristics of prediction model building dataset and validation dataset.

Supplemental Table S2. Sequencing result of prediction model building dataset (111 samples). "Average" represents the average value of each statistical data among the 111 samples, while "Range" represents the minimum and maximum values of the data among the 111 samples.

Supplemental Table S3. Statistical analysis result of miRNAs included in the prediction model building dataset.

Supplemental Table S4. The performance of model training.

Supplemental Figure S1.

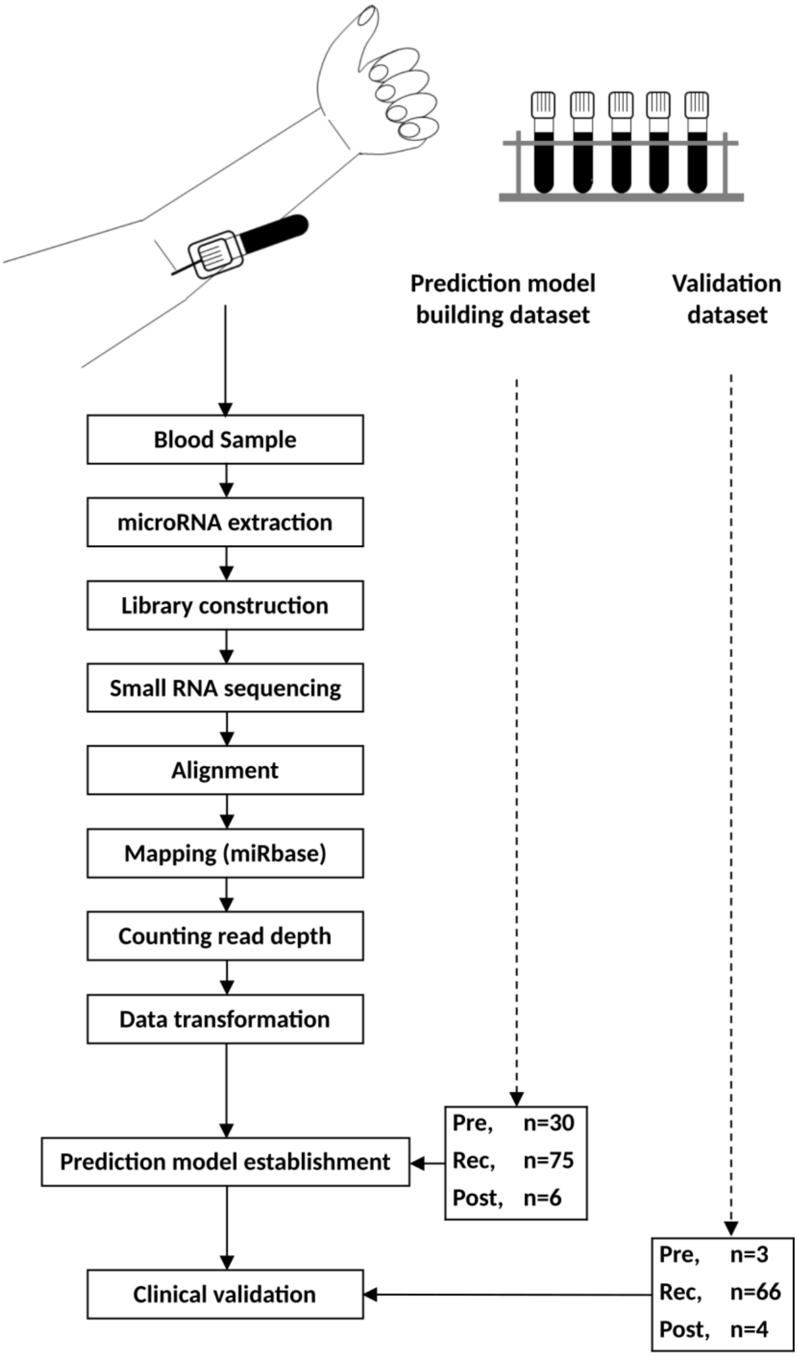

Supplemental Table S1. Clinical characteristics of prediction model building dataset and validation dataset

Parameter	Prediction model building dataset			Validation dataset		
	Pre (n=30)	Rec (n=75)	Post (n=6)	Pre (n=3)	Rec (n=67)	Post (n=4)
Age (years)						
Median (range)	36.9 (30 - 44)	37.8 (30 - 50)	36.0 (28 - 39)	37.7 (34 - 41)	36.6 (29 - 44)	36.0 (35 - 37)
BMI (kg/m^2)						
Median (range)	23.4 (18.3 - 35.3)	23.1 (18.3 - 38.3)	23.4 (21.1 - 25)	22.0 (19.2 - 23.6)	22.9 (18.2 - 35.3)	25.0 (24.5 - 25.5)

Supplemental Table S2. Sequencing result of prediction model building dataset (111 samples)

Item	Average	Range
Total reads	8,000,795	(5,459,122 - 10,043,952)
miRNA reads	395,433	(15,567 - 1,675,377)
miRNA% (miRNA reads/Total reads)	4.8%	(0.3% - 18.7%)
Detectable miRNAs (read counts > 20)	135	(40 - 263)

Supplemental Table S3. Statistical analysis result of miRNAs included in the prediction model building dataset

miRNA	miRNA expression level						ANOVA		Post Hoc Tukey HSD								
	Pre-receptive (Pre)		Receptive (Rec)		Post-receptive (Post)				Pre:Rec			Pre:Post			Rec:Post		
	Mean	SD	Mean	SD	Mean	SD	F score	p-value	Q score	p-value	FC	Q score	p-value	FC	Q score	p-value	FC
hsa-let-7b-5p	15.32	0.57	14.95	0.52	14.38	0.39	25.17	< 0.001	4.46	0.005	1.30	11.16	< 0.001	1.92	6.69	< 0.001	1.48
hsa-miR-3184-3p	13.52	0.68	13.13	0.53	12.73	0.43	16.70	< 0.001	4.17	0.010	1.30	8.56	< 0.001	1.72	4.39	0.006	1.32
hsa-miR-423-5p	13.52	0.68	13.13	0.53	12.73	0.43	16.70	< 0.001	4.17	0.010	1.30	8.56	< 0.001	1.72	4.39	0.006	1.32
hsa-let-7g-5p	14.65	0.37	14.39	0.41	13.95	0.31	23.45	< 0.001	4.06	0.013	1.20	10.94	< 0.001	1.63	6.88	< 0.001	1.36
hsa-miR-4635	12.67	1.28	12.21	1.27	11.95	1.00	3.75	0.025	2.28	0.243	1.38	3.53	0.035	1.65	1.26	0.649	1.19
hsa-miR-4685-5p	14.18	1.33	13.70	1.25	13.42	0.85	4.06	0.019	2.34	0.225	1.39	3.75	0.023	1.69	1.42	0.577	1.22
hsa-miR-4771	12.86	1.27	12.32	1.21	12.20	0.99	4.61	0.011	2.71	0.136	1.45	3.34	0.049	1.58	0.63	0.896	1.09
hsa-miR-92a-3p	10.63	0.59	10.21	0.71	10.07	0.83	9.28	< 0.001	3.76	0.023	1.34	5.08	0.001	1.48	1.33	0.617	1.11
hsa-miR-320a-3p	13.99	0.77	13.55	0.60	13.42	0.64	11.31	< 0.001	4.20	0.009	1.36	5.44	< 0.001	1.49	1.24	0.655	1.10
hsa-miR-1180-3p	7.44	0.53	7.08	0.55	6.87	0.46	12.61	< 0.001	4.15	0.010	1.28	6.56	< 0.001	1.48	2.41	0.207	1.16
hsa-miR-26a-5p	13.67	0.57	13.30	0.73	13.15	0.31	7.80	0.001	3.39	0.045	1.29	4.82	0.002	1.43	1.42	0.574	1.11
hsa-miR-10a-5p	7.85	0.91	7.32	0.82	7.35	0.59	9.12	< 0.001	3.97	0.015	1.45	3.73	0.024	1.42	0.24	0.984	-2.01
hsa-miR-26b-5p	11.28	0.46	11.00	0.72	10.85	0.62	4.88	0.008	2.60	0.161	1.21	4.04	0.013	1.34	1.44	0.564	1.11
hsa-miR-484	8.81	0.46	8.45	0.71	8.40	0.57	7.31	0.001	3.47	0.039	1.28	3.93	0.016	1.33	0.46	0.942	1.03
hsa-miR-23a-3p	9.36	0.75	8.84	0.83	8.95	0.70	8.99	< 0.001	3.97	0.015	1.43	3.13	0.072	1.32	0.85	0.821	-2.02
hsa-miR-451a	11.41	0.49	11.04	0.67	11.03	0.95	7.50	0.001	3.57	0.033	1.30	3.61	0.030	1.30	0.04	1.000	1.00
hsa-miR-193a-5p	8.20	0.82	7.91	0.78	8.37	0.84	4.62	0.011	2.23	0.257	1.22	1.28	0.638	-2.03	3.51	0.036	-2.08
hsa-miR-375-3p	8.32	1.23	7.68	1.15	7.82	0.60	6.96	0.001	3.50	0.038	1.56	2.75	0.129	1.42	0.75	0.858	-2.03
hsa-miR-320b	11.95	1.17	11.47	0.85	11.55	0.94	5.63	0.004	3.14	0.070	1.40	2.60	0.160	1.32	0.54	0.923	-2.01
hsa-miR-320d	10.64	1.23	10.12	0.93	10.27	0.78	5.82	0.003	3.20	0.063	1.44	2.32	0.232	1.30	0.88	0.807	-2.02
hsa-miR-143-3p	10.54	0.54	9.85	0.76	10.17	1.17	18.74	< 0.001	5.71	< 0.001	1.61	3.12	0.072	1.30	2.59	0.161	-2.04
hsa-miR-181b-5p	8.26	0.74	7.74	0.68	7.93	0.58	12.14	< 0.001	4.62	0.004	1.43	2.93	0.098	1.25	1.70	0.455	-2.03
hsa-miR-23b-3p	7.69	0.91	7.24	0.91	7.40	0.75	5.43	0.005	3.09	0.076	1.37	2.02	0.329	1.23	1.08	0.727	-2.03
hsa-miR-320c	11.29	1.17	10.83	0.91	11.02	0.97	4.75	0.010	2.88	0.105	1.38	1.72	0.445	1.21	1.16	0.689	-2.02
hsa-miR-30a-3p	7.26	0.60	6.92	0.69	7.05	0.71	5.68	0.004	3.16	0.068	1.27	1.95	0.353	1.16	1.20	0.671	-2.03
hsa-miR-12116	9.45	1.39	8.83	1.54	9.27	1.30	3.52	0.031	2.54	0.174	1.54	0.76	0.854	1.14	1.78	0.420	-2.07
hsa-miR-181a-5p	8.72	0.79	8.33	0.80	8.53	0.46	5.39	0.005	3.05	0.080	1.30	1.46	0.556	1.14	1.59	0.499	-2.03
hsa-miR-199a-3p	9.85	0.73	9.41	0.70	9.75	0.72	9.05	< 0.001	3.83	0.020	1.36	0.88	0.809	1.07	2.95	0.096	-2.05
hsa-miR-199b-3p	9.85	0.73	9.41	0.70	9.75	0.72	9.05	< 0.001	3.83	0.020	1.36	0.88	0.809	1.07	2.95	0.096	-2.05
hsa-miR-378c	8.16	0.87	7.83	0.97	8.13	1.17	2.80	0.063	2.07	0.311	1.25	0.14	0.994	1.02	1.92	0.363	-2.05
hsa-miR-339-5p	8.02	1.14	7.63	1.20	8.07	1.04	2.98	0.053	2.07	0.309	1.32	0.22	0.987	-2.01	2.29	0.239	-2.08
hsa-miR-5585-5p	7.70	1.60	7.37	1.35	6.10	0.85	7.71	0.001	1.37	0.598	1.26	6.51	< 0.001	3.04	5.14	0.001	2.40
hsa-miR-629-5p	9.85	1.60	9.51	1.62	8.27	1.39	6.80	0.001	1.31	0.626	1.26	6.11	< 0.001	2.99	4.80	0.002	2.37
hsa-miR-3960	10.30	1.08	10.50	1.32	9.28	0.73	7.94	< 0.001	0.96	0.774	-2.03	5.15	0.001	2.03	6.12	< 0.001	2.32
hsa-miR-191-5p	13.42	0.38	13.18	0.38	12.55	0.27	38.64	< 0.001	4.10	0.011	1.19	14.49	< 0.001	1.83	10.39	< 0.001	1.54
hsa-miR-30e-3p	7.93	0.46	7.75	0.77	7.22	0.43	7.73	0.001	1.61	0.493	1.13	6.51	< 0.001	1.64	4.90	0.002	1.45
hsa-let-7d-5p	12.24	0.35	12.16	0.34	11.57	0.51	25.22	< 0.001	1.23	0.658	1.05	11.46	< 0.001	1.59	10.23	< 0.001	1.51
hsa-miR-130b-3p	8.75	0.49	8.54	0.46	8.13	0.33	13.26	< 0.001	2.84	0.112	1.16	8.34	< 0.001	1.54	5.49	< 0.001	1.33
hsa-miR-744-5p	7.57	0.67	7.36	0.56	6.97	0.47	7.75	0.001	2.21	0.264	1.16	6.36	< 0.001	1.51	4.15	0.010	1.31
hsa-let-7f-5p	13.72	0.44	13.64	0.43	13.17	0.51	11.25	< 0.001	1.12	0.710	1.06	7.76	< 0.001	1.47	6.64	< 0.001	1.39
hsa-miR-151a-5p	14.18	0.50	14.09	0.48	13.67	0.18	8.23	< 0.001	1.22	0.664	1.07	6.70	< 0.001	1.43	5.47	< 0.001	1.34
hsa-let-7e-5p	8.19	0.59	8.18	0.58	7.70	0.85	5.39	0.005	0.10	0.997	1.01	5.05	0.001	1.41	4.95	0.002	1.40
hsa-miR-182-5p	8.32	0.62	8.30	0.60	7.88	0.74	3.92	0.021	0.16	0.993	1.01	4.36	0.007	1.35	4.19	0.009	1.33
hsa-miR-421	7.89	0.33	7.91	0.45	7.48	0.36	8.56	< 0.001	0.31	0.974	-2.00	6.04	< 0.001	1.32	6.35	< 0.001	1.34
hsa-miR-486-5p	15.10	0.42	14.95	0.54	14.68	0.38	5.05	0.007	1.83	0.401	1.11	5.11	0.001	1.33	3.28	0.055	1.20
hsa-miR-20b-5p	9.58	0.53	9.59	0.60	9.18	0.30	4.28	0.015	0.17	0.992	-2.00	4.31	0.007	1.32	4.48	0.005	1.33
hsa-let-7a-5p	15.65	0.35	15.66	0.34	15.27	0.51	9.85	< 0.001	0.12	0.996	-2.00	6.64	< 0.001	1.31	6.76	< 0.001	1.31
hsa-miR-21-5p	9.42	0.55	9.37	0.51	9.85	0.36	6.92	0.001	0.52	0.929	1.03	5.21	0.001	-2.06	5.73	< 0.001	-2.07
hsa-miR-29a-3p	9.89	0.66	9.92	0.64	10.37	0.71	4.10	0.018	0.27	0.980	-2.00	4.51	0.005	-2.07	4.24	0.008	-2.06
hsa-miR-122-5p	14.40	1.19	14.47	1.40	15.67	1.39	6.83	0.001	0.30	0.975	-2.01	5.80	< 0.001	-2.13	5.50	< 0.001	-2.12
hsa-miR-122b-3p	14.35	1.18	14.43	1.39	15.62	1.37	6.87	0.001	0.34	0.968	-2.01	5.84	< 0.001	-2.13	5.50	< 0.001	-2.12
hsa-miR-1285-3p	10.06	0.77	9.77	1.09	9.27	0.89	4.61	0.011	1.78	0.420	1.22	4.87	0.002	1.73	3.09	0.076	1.42
hsa-let-7i-5p	14.19	0.27	13.94	0.28	13.70	0.43	24.65	< 0.001	5.17	0.001	1.18	10.28	< 0.001	1.40	5.11	0.001	1.18
hsa-miR-486-3p	14.99	0.41	14.86	0.53	14.62	0.37	4.13	0.017	1.62	0.486	1.09	4.63	0.003	1.29	3.01	0.086	1.18
hsa-miR-98-5p	10.09	0.34	9.94	0.35	9.73	0.30	8.64	< 0.001	2.76	0.126	1.11	6.41	< 0.001	1.28	3.65	0.028	1.15
hsa-miR-150-5p	8.77	0.70	8.56	0.89	8.40	0.46	1.98	0.140	1.56	0.514	1.15	2.78	0.124	1.29	1.22	0.664	1.12
hsa-miR-339-3p	7.57	0.54	7.42	0.48	7.23	0.46	3.76	0.025	1.86	0.389	1.11	4.20	0.009	1.26	2.34	0.226	1.14
hsa-miR-185-5p	14.18	0.44	14.00	0.64	13.87	0.24	2.88	0.058	1.87	0.386	1.13	3.36	0.048	1.24	1.50	0.542	1.10
hsa-let-7c-5p	11.32	0.51	11.32	0.62	11.05	0.24	1.90	0.152	0.00	< 0.001	1.00	2.96	0.094	1.21	2.95	0.094	1.21
hsa-miR-29c-3p	8.79	0.64	8.87	0.51	9.17	0.62	3.15	0.045	0.84	0.822	-2.01	4.16	0.010	-2.06	3.31	0.052	-2.05

hsa-miR-144-3p	10.59	0.61	10.77	0.61	10.97	0.64	3.27	0.040	1.81	0.409	-2.02	3.83	0.020	-2.05	2.03	0.325	-2.03
hsa-miR-142-5p	8.36	0.57	8.50	0.63	8.72	0.47	2.72	0.068	1.47	0.552	-2.02	3.66	0.028	-2.06	2.19	0.270	-2.04
hsa-miR-625-5p	7.11	0.71	7.33	0.86	7.53	0.41	2.53	0.082	1.69	0.457	-2.04	3.24	0.059	-2.08	1.55	0.516	-2.04
hsa-miR-3135b	7.52	1.43	7.83	1.20	7.18	0.55	2.93	0.056	1.50	0.538	-2.06	1.71	0.451	1.27	3.21	0.062	1.56
hsa-miR-103a-3p	14.12	0.28	14.15	0.54	13.85	0.39	3.19	0.043	0.36	0.964	-2.00	3.53	0.035	1.21	3.89	0.018	1.23
hsa-miR-103b	14.12	0.28	14.15	0.54	13.85	0.39	3.19	0.043	0.36	0.964	-2.00	3.53	0.035	1.21	3.89	0.018	1.23
hsa-miR-93-5p	13.54	0.42	13.57	0.43	13.32	0.26	3.02	0.051	0.45	0.945	-2.00	3.33	0.051	1.17	3.78	0.022	1.19
hsa-miR-361-5p	7.68	0.61	7.43	0.63	7.87	0.32	6.61	0.002	2.55	0.172	1.19	1.86	0.387	-2.03	4.41	0.006	-2.08
hsa-miR-378a-3p	9.26	0.74	9.04	0.77	9.43	0.72	3.45	0.033	1.81	0.408	1.17	1.43	0.570	-2.03	3.24	0.059	-2.06
hsa-miR-1260b	8.18	1.73	7.80	1.72	7.70	0.99	1.14	0.323	1.36	0.601	1.30	1.74	0.437	1.39	0.37	0.962	1.07
hsa-miR-342-3p	8.56	1.24	8.20	1.23	8.17	1.06	2.00	0.138	1.83	0.401	1.29	1.99	0.339	1.31	0.16	0.993	1.02
hsa-miR-28-3p	7.92	0.68	7.69	0.76	7.58	0.86	2.50	0.085	1.90	0.374	1.17	2.79	0.121	1.26	0.90	0.801	1.08
hsa-miR-8485	9.09	1.25	8.97	1.32	8.82	1.29	0.33	0.717	0.56	0.917	1.09	1.29	0.635	1.21	0.73	0.865	1.11
hsa-miR-107	14.09	0.29	14.12	0.59	13.85	0.39	2.20	0.113	0.36	0.964	-2.00	2.87	0.107	1.18	3.24	0.059	1.20
hsa-miR-151a-3p	9.30	0.50	9.08	0.51	9.17	0.45	4.24	0.016	2.73	0.133	1.17	1.63	0.482	1.10	1.09	0.719	-2.01
hsa-miR-3529-3p	7.93	0.62	7.98	0.66	7.78	0.42	0.79	0.453	0.46	0.944	-2.01	1.44	0.567	1.11	1.90	0.374	1.14
hsa-miR-7-5p	7.93	0.62	7.98	0.66	7.78	0.42	0.79	0.453	0.46	0.944	-2.01	1.44	0.567	1.11	1.90	0.374	1.14
hsa-miR-425-5p	8.84	0.41	8.70	0.47	8.70	0.46	2.16	0.118	1.92	0.367	1.10	1.95	0.353	1.11	0.04	1.000	1.00
hsa-miR-25-3p	10.19	0.42	10.15	0.38	10.07	0.21	0.79	0.453	0.67	0.884	1.03	2.05	0.317	1.09	1.38	0.593	1.06
hsa-miR-126-3p	12.64	0.42	12.43	0.48	12.53	0.53	4.52	0.012	2.80	0.120	1.16	1.38	0.591	1.08	1.41	0.578	-2.01
hsa-miR-30d-5p	7.81	0.51	7.57	0.44	7.72	0.34	6.20	0.002	3.24	0.059	1.18	1.23	0.662	1.06	2.01	0.332	-2.03
hsa-miR-20a-5p	10.22	0.51	10.11	0.68	10.18	0.42	0.67	0.510	1.06	0.733	1.08	0.36	0.964	1.03	0.70	0.874	-2.01
hsa-miR-106b-5p	7.19	0.58	7.12	0.61	7.15	0.39	0.33	0.722	0.76	0.853	1.05	0.42	0.953	1.03	0.34	0.968	-2.01
hsa-miR-574-5p	13.05	0.63	12.92	0.54	13.00	0.36	1.20	0.303	1.43	0.572	1.09	0.57	0.916	1.04	0.86	0.816	-2.01
hsa-miR-660-5p	8.47	0.54	8.30	0.53	8.42	0.50	2.37	0.096	1.99	0.339	1.13	0.65	0.892	1.04	1.34	0.609	-2.02
hsa-miR-181a-3p	9.27	1.23	9.06	1.37	9.22	0.85	0.65	0.523	1.03	0.746	1.16	0.27	0.980	1.04	0.76	0.853	-2.02
hsa-miR-223-3p	10.45	0.68	10.23	0.89	10.43	0.20	1.90	0.152	1.70	0.454	1.17	0.11	0.997	1.01	1.59	0.501	-2.03
hsa-miR-130a-3p	10.41	0.38	10.39	0.48	10.37	0.17	0.12	0.888	0.38	0.961	1.02	0.68	0.879	1.03	0.30	0.975	1.01
hsa-miR-139-5p	8.29	0.64	8.07	0.63	8.28	0.70	3.20	0.043	2.19	0.270	1.17	0.08	0.998	1.01	2.11	0.298	-2.04
hsa-miR-24-3p	9.60	0.73	9.49	0.58	9.63	0.73	0.90	0.409	1.07	0.731	1.08	0.31	0.974	-2.00	1.38	0.594	-2.02
hsa-miR-3074-5p	9.55	0.74	9.42	0.59	9.58	0.71	1.13	0.326	1.19	0.676	1.09	0.36	0.965	-2.01	1.55	0.517	-2.02
hsa-miR-10b-5p	7.73	0.82	7.57	0.78	7.82	0.73	1.43	0.240	1.27	0.643	1.12	0.65	0.891	-2.01	1.92	0.367	-2.05
hsa-miR-6873-3p	10.11	1.83	9.97	1.60	10.20	1.65	0.25	0.781	0.49	0.935	1.10	0.32	0.973	-2.01	0.81	0.835	-2.03
hsa-miR-126-5p	11.73	0.47	11.62	0.45	11.83	0.53	2.41	0.093	1.41	0.579	1.08	1.41	0.577	-2.01	2.83	0.115	-2.03
hsa-miR-15a-5p	10.67	0.55	10.83	0.50	10.82	0.42	2.09	0.125	1.91	0.370	-2.02	1.74	0.438	-2.02	0.17	0.992	1.01
hsa-miR-140-3p	7.58	0.57	7.81	0.52	7.73	0.31	4.35	0.014	2.77	0.125	-2.04	1.82	0.405	-2.03	0.95	0.779	1.06
hsa-miR-128-3p	7.02	0.54	7.22	0.53	7.18	0.39	3.02	0.051	2.30	0.237	-2.04	1.88	0.380	-2.03	0.42	0.953	1.02
hsa-miR-1290	11.05	0.92	10.82	0.88	11.20	1.25	2.33	0.099	1.54	0.520	1.17	1.02	0.750	-2.02	2.57	0.167	-2.05
hsa-miR-532-5p	8.26	0.32	8.31	0.47	8.42	0.42	0.95	0.388	0.74	0.861	-2.01	2.25	0.253	-2.03	1.51	0.535	-2.02
hsa-miR-151b	8.78	0.70	8.73	0.64	8.93	0.59	0.85	0.430	0.47	0.940	1.04	1.48	0.547	-2.02	1.96	0.351	-2.03
hsa-miR-15b-5p	11.20	0.38	11.40	0.49	11.42	0.23	4.50	0.012	2.74	0.130	-2.02	2.96	0.094	-2.03	0.21	0.988	-2.00
hsa-miR-30e-5p	8.92	0.56	8.94	0.53	9.15	0.31	1.43	0.241	0.22	0.987	-2.00	2.70	0.139	-2.04	2.48	0.188	-2.03
hsa-miR-16-5p	18.54	0.40	18.72	0.39	18.80	0.24	5.61	0.004	2.84	0.112	-2.01	4.19	0.009	-2.02	1.35	0.608	-2.01
hsa-miR-7847-3p	10.82	1.52	10.51	1.45	11.07	0.87	1.85	0.159	1.31	0.624	1.24	1.08	0.727	-2.03	2.39	0.212	-2.07
hsa-miR-1246	8.84	1.17	8.55	1.31	9.20	1.01	2.24	0.109	1.22	0.664	1.22	1.49	0.545	-2.06	2.71	0.137	-2.11
hsa-miR-1303	8.55	0.91	8.26	1.41	8.92	0.78	2.41	0.093	1.30	0.630	1.23	1.62	0.486	-2.06	2.92	0.100	-2.11
hsa-miR-4510	7.52	1.74	7.42	1.79	8.04	0.95	0.88	0.415	0.33	0.971	1.07	1.73	0.440	-2.10	2.06	0.314	-2.12

FC: fold change

Supplemental Table S4. The performance of model training

Model	Purpose	Data input	Factor	Prediction model	Pre-receptive		Receptive		Post-receptive	
					Sensitivity	Precision	Sensitivity	Precision	Sensitivity	Precision
1	Post-receptive identification	Normalized read counts and probability	Age	Logistic Regression	0.0%	0.0%	80.6%	94.7%	100.0%	100.0%
2	Receptive identification	Normalized read counts and probability	Age	Random Forest	66.7%	50.0%	97.0%	98.5%	-	-

Additional information for model building and selection

Data Augmentation:

By repeatedly randomly sampling five million reads from the NGS data of the same specimen, we generated in silico duplications as a means of data augmentation. The purpose of this sampling was to intentionally create in silico duplications within the dataset. This step is able to address potential limitations associated with dataset size and diversity. By generating in silico duplications, a larger and more balanced dataset can increase the robustness and representativeness and improve the statistical power and reliability of subsequent computational tasks and analyses.

Normalization:

The total count of miRNA is calculated by summing the values of total miRNAs for each sample. The ratio of each miRNA is calculated by dividing the read counts of each miRNA by the total counts of miRNA. The resulting values are multiplied by 1,000,000 to obtain a ratio. After data normalization, we also compute the log2 transformation of a given final normalized value.

Subject information:

In addition to miRNA expression level, the patients' age and BMI were also collected as features for model training.

Transform miRNA expression levels into probability:

First, we calculate the mean and standard deviation of normalized miRNA expression levels from the model building dataset for pre-receptive, receptive and post-receptive statuses. Then, we build the normal distribution for different statuses. Finally, we take normalized miRNA expression level of each miRNA into the normal distribution to calculate the probability of each different statuses by the following formula..

- X as the miRNA expression value,
- μ as the mean of the distribution for a specific classification,
- σ as the standard deviation of the distribution for that classification,

The probability density function (PDF) of the normal distribution is given by:

$$P(X) = \frac{1}{\sigma\sqrt{2\pi}} e^{-\frac{(X-\mu)^2}{2\sigma^2}}$$

Model training and hyper-parameter turning:

We performed 10-fold cross-validation for hyper-parameter turning by using the Logistic Regression, Random Forest Classifier and k-Nearest Neighbors (KNN). Model selection was determined according to the returned evaluation metrics, such as

accuracy, precision, recall, and F1-score. The hyperparameter grid for each classifier we considered is detailed below.

1. **Hyperparameter grid for Logistic regression:** The following hyperparameters is used in this study. In this case, it is set to L1 regularization, which involves adding the absolute values of the coefficients as a penalty term to the optimization objective. The parameter 'C' represents the inverse of regularization strength. In logistic regression, the solver is an optimization algorithm used to find the optimal coefficients. It is set to 10,000 iterations.
2. **Hyperparameter grid for random forest classifier:**
   The grid search cross-validation of random forest explores various combinations of hyperparameters, including: number of trees in the forest, the maximum number of features considered for splitting a node and the maximum depth of the and criterion use gini index.
3. **Hyperparameter grid for k-Nearest Neighbors (kNN) optimization:**
   The hyperparameter under consideration is the number of neighbors.

The grid search for each classifier is orchestrated through the cuml library, ensuring a comprehensive exploration of hyperparameter space.

Model selection:

Based on the result, we combined 2 models to create the final predictive model for 2-step classification. Model 1 has a sensitivity of 100.0% and precision of 100.0% in identifying post-receptive samples. Model 2 has a sensitivity of 97.0% and precision of 98.5% in identifying receptive samples (Table S4). The process for determining the endometrial receptivity status involved utilizing both models. The first step was to determine if the endometrial status is post-receptive using Model 1. If the sample is not post-receptive, the second step utilizes Model 2 to determined whether the sample was pre-receptive or receptive.
